# Acute massive congestive ischaemic colitis related to inferior mesenteric arteriovenous malformation

**DOI:** 10.1259/bjrcr.20150275

**Published:** 2016-01-19

**Authors:** Bruno Coulier, Jean-François De Wispelaere, Isabelle Bueres, Frédéric Pierard, Fabrice Cedric Deprez, Philippe Maldague, Marc van Hoof, Isabelle Gielen

**Affiliations:** ^1^ Department of Diagnostic Radiology, Clinique St Luc, Bouge (Namur), Belgium; ^2^ Departments of Diagnostic and Interventional Radiology, CHU Mont-Godinne, Yvoir, Belgium; ^3^ Department of Gastroenterology, Clinique St Luc, Bouge (Namur), Belgium; ^4^ Department of Visceral Surgery, Clinique St Luc, Bouge (Namur), Belgium; ^5^ Department of Pathology, Institute of Pathology and Genetics, Gosselies, Belgium

## Abstract

We report a very rare case of acute congestive ischaemic colitis of the left colon caused by brutal decompensation of an uncommon arteriovenous malformation (AVM) in the territory of the inferior mesenteric artery (IMA) in a 45-year-old male patient. The patient presented with severe abdominal pain in the left iliac fossa and abundant mucoid stools. The diagnosis of congestive colitis was made by optical colonoscopy but the full diagnosis of the responsible AVM in the IMA territory was made by contrast-enhanced multidetector CT scan combined with colour Doppler ultrasound. Two successive attempts at selective embolization failed to resolve the symptoms and finally, extensive surgery was necessary. The complete imaging findings of the case are presented and the characteristic features of uncommon AVMs and fistulas of the IMA territory are briefly reviewed.

## Introduction

Ischaemic colitis (IC) is the most prevalent type of gastrointestinal ischaemia (50–60% of all episodes).^1,2^ Its real incidence is probably underestimated because many patients suffer only mild or transient damage that remains undiagnosed. IC has a female gender predilection and mostly affects elderly patients, with 90% of them being over 60 years of age. Patients with IC frequently present with co-morbid conditions.

The aetiologies of IC are numerous and lead to a diminished perfusion of the colon, which may result in reversible mucosal injury or even full-thickness necrosis. Fortunately, most cases (80–85%) only result in non-gangrenous segmental IC and resolve with medical management, without a specific cause being found.^2^

IC is frequently classified as occlusive or non-occlusive. Occlusive IC can not only be attributed to thrombosis or emboli in large vessels, numerous and various chronic diseases of small vessels, trauma, various surgical procedures and iatrogenic causes (endoscopy, colonoscopy, barium enema, etc.) but also to venous causes resulting from venous thrombosis or venous hypertension.^2^ Non-occlusive causes comprise numerous life-threatening situations resulting in systemic hypoperfusion, situations of colonic dilatation or obstruction, iatrogenic causes related to a long list of drugs and long-distance running.

We hereby report a typical but very unusual case of acute congestive IC related to the decompensation of a very uncommon arteriovenous malformation (AVM) in the inferior mesenteric artery (IMA) territory.

## Clinical presentation

A 45-year-old male patient was admitted to the emergency department with a 24-h history of heavy abdominal pain. The patient had previously experienced a prolonged episode of constipation and had been treated with laxatives. He was extremely obese and had a previous history of severe ischaemic cardiopathy complicated by episodes of ventricular tachycardia and auricular fibrillation. At admission, the pain was focalized in the hypogastrium and the left iliac fossa. These areas were painful to palpation with rebound. The patient also presented a small ecchymotic umbilical hernia. He was apyretic and had recurrent episodes of abundant mucoid stools. There was no rectal bleeding or melena. Laboratory tests were unremarkable except for a mild inflammatory syndrome with a C-reactive protein protein level ranging from 92 mg l^–1^ at admission to 215 mg l^–1^ 4 days later.

## Investigations and imaging findings

An unenhanced abdominal multidetector CT (MDCT; not illustrated) scan was first performed because the patient was allergic to iodinated contrast media. A marked homogeneously circumferential hypodense thickening of a very long segment of the left colon extending from the descending colon to the rectosigmoid junction was found. Prominent vessels and massive fat stranding were depicted in the mesosigmoid fat. A small amount of ascites were present. There were no colonic diverticula and the diagnosis of colonic diverticulitis was excluded. The first retained diagnosis was IC or inflammatory colonic disease.

**Figure 1.**
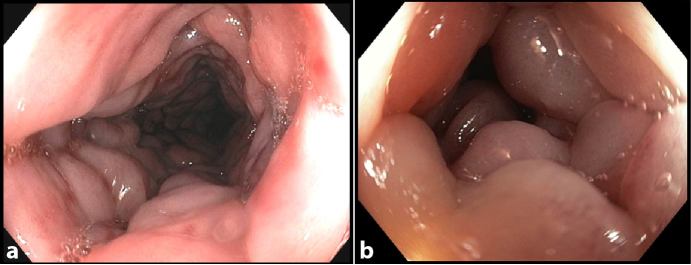
Optical colonoscopy (a, b) of the sigmoid colon shows the extremely oedematous, cobblestone appearance of the colonic mucosa causing narrowing of the lumen, especially at the level of the descending colon (b). No ulceration or blood was found.

Careful optical colonoscopy (Figure 1) was performed the same day and demonstrated an extremely oedematous, cobblestone appearance of the colonic mucosa causing narrowing of the lumen, especially at the level of the descending colon. There was neither blood nor ulceration. Limited biopsies were performed because the musosa bleed easily. Histopathology demonstrated congestive and oedematous colonic mucosa with no trace of acute or chronic inflammatory process and no sign of infection.

**Figure 2.**
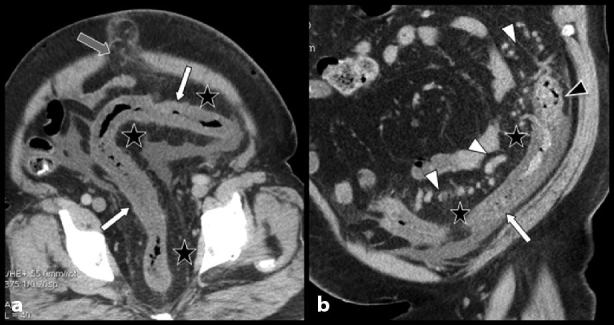
Contrast-enhanced abdominal CT images. Oblique (a) and coronal oblique (b) multiplanar reconstructions show severe but homogeneously circumferential hypodense thickening of a rather long segment of the left colon extending from the descending colon to the rectosigmoid junction (white arrows) and fat stranding of the umbilical hernia (grey arrow). There is a sharp cutoff of the thickening at the level of the left phrenicocolic ligament. Diffuse congestive fat stranding is associated with the mesosigmoid and the greater omentum (black stars). Unusually large and serpiginous varicosities are found not only in the mesosigmoid fat (white arrowheads on b) but also in the colonic wall (black arrowhead on b). Ascites are visible in the sigmoid and the right iliac fossa.

**Figure 3.**
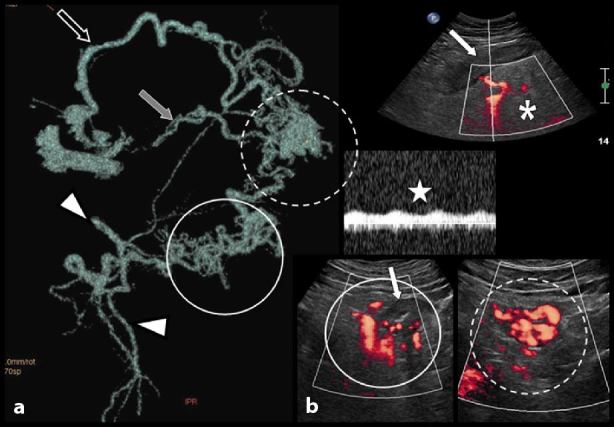
Contrast-enhanced abdominal CT images. Selective volume rendering reconstruction of the massive serpiginous mesosigmoid varicosities (a). They converge to a dilated vein running through the transverse mesocolon to penetrate the venous splenoportal confluence at right angle (black arrow). An accessory venous drainage also runs to the left renal vein (grey arrow). The inferior mesenteric vein is constitutionally absent in this patient (not illustrated). The inferior mesenteric artery is also visible (white arrowheads). Complementary colour Doppler ultrasound was performed (b). Ultrasound palpation of the left iliac fossa was very painful and confirmed a very thickened hypoechoic sigmoid colon (white arrows) running through a diffusely painful and uncompressible hyperechoic inflammatory mesocolonic fat (white asterisk). Colour Doppler confirmed major varicosities not only along the thickened colonic wall (full circle) but also within the colonic wall (dotted circle). The Doppler spectrum of these varicosities contains a pulsatile arterial component (white star) strongly suggesting an arteriovenous communication.

A complementary contrast-enhanced abdominal MDCT (64 row MDCT, 140 kV, auto mA modulation, 0.6 s/rot, 55 mm/rot, thickness 1.2 mm, pitch 1.375 : 1, reconstruction spacing 0.7 mm) was performed 48 h later after premedicating with oral steroid antiallergic (Figures 2 and 3). Enhancement of the vessels was suboptimal, probably because of the morbid obesity and cardiac insufficiency of the patient. Nevertheless, the homogeneously circumferential hypodense thickening of the left colon was confirmed and better imaged. There was a sharp cutoff of the thickening at the level of the left phrenicocolic ligament, and massive congestive fat stranding of the mesosigmoid, the greater omentum and the umbilical hernia. The prominent vessels were identified as unusually large and serpiginous varicosities were identified running not only through the mesosigmoid fat but also within the colonic wall, especially at the level of the phrenicocolic ligament.

These varicosities converged to a dilated vein running through the transverse mesocolon to penetrate the venous splenoportal confluence at a right angle. An accessory drainage also joined the left renal vein. The inferior mesenteric vein was constitutionally absent.

**Figure 4.**
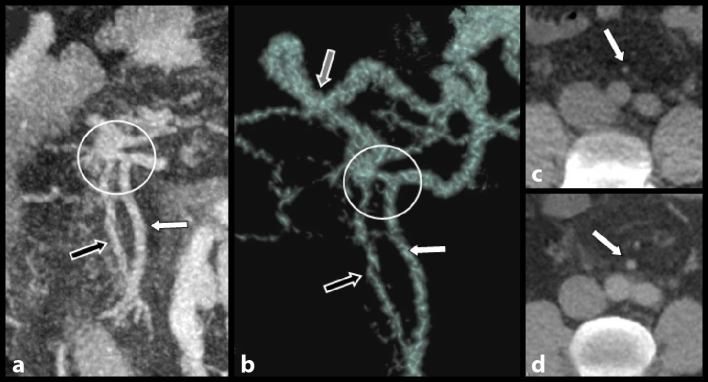
Contrast-enhanced abdominal CT images. Details of the maximum intensity projection view (a) and the volume rendering view (b) show the superior rectal artery (white arrows), the normal superior rectal vein (black arrows) and the presence of an indisputable arteriovenous communication (white circles). It is noteworthy that the caliber of the rectal vein abruptly increases just distal to this anomaly (grey arrow on b). Moreover, the caliber of the inferior mesenteric artery also appears considerably increased (white arrow on d) when compared with a view obtained 4 years ago (white arrow on c).

Scrupulous analysis of volume rendering and maximum intensity projection clearly diagnosed an arteriovenous communication in the distal mesosigmoid (Figure 4). There was an abrupt increase in the caliber of the rectal vein just distal to the malformation. Also, there was considerable increase in the caliber of the IMA when compared with a view obtained 4 years ago. A colour Doppler ultrasound (Figure 3b) confirmed major varicosities not only along the thickened colonic wall but also within the wall. The Doppler spectrum of these varicosities showed a pulsatile arterial component, reinforcing the hypothesis of an AVM.

## Treatment

**Figure 5.**
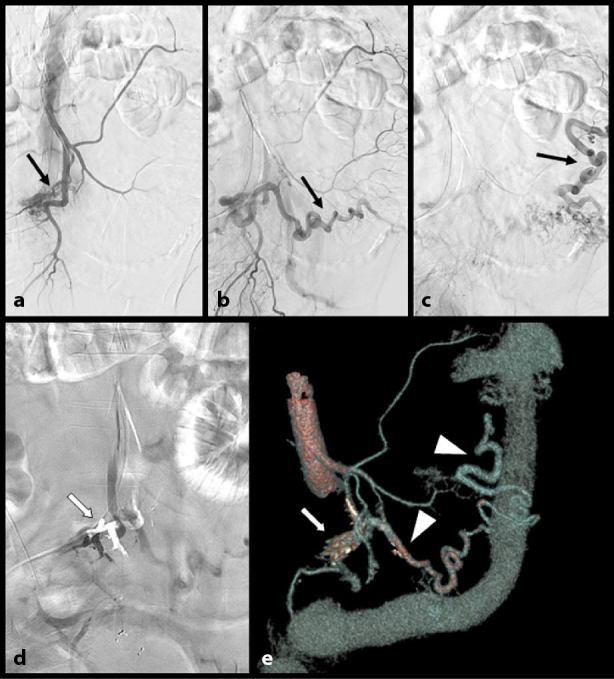
Selective arteriography (a–c) of the inferior mesenteric artery confirms the diagnosis of an arteriocapillary type malformation (black arrows) with a rather low flow. Careful embolization with ethylene vinyl alcohol copolymer (ONYX^®^, Micro Therapeutic Inc., Irvine, CA) was performed (white arrows on d and e). Nevertheless, 3 days later, important symptoms persisted. A new angio-CT scan with volume rendering views (e) confirmed the persistence of an active arteriovenous fistula (white arrowheads).

4 days after admission, a decision to first treat the patient with selective embolization was proposed. Selective arteriography (Figure 5) of the IMA first confirmed the diagnosis of an arteriocapillary malformation with a rather low flow. Careful embolization with ethylene vinyl alcohol copolymer (ONYX^®^ 18 liquid embolic system, Micro Therapeutic Inc., Irvine, CA) was performed. ONYX^®^ was preferred to the traditional glue or coils to allow a more distal and controlled embolization. The goal was to protect the arteries of larger caliber and avoid a dramatic necrosis of the sigmoid and, especially, the rectum.

Nevertheless, 3 days later, the important symptoms persisted and a new contrast-enhanced MDCT confirmed the persistence of an active arteriovenous communication, which was probably owing to the presence of untreated areas (Figure 5e).

A second selective embolization session was performed 2 days later (not illustrated). The patient presented a significant transitory improvement in symptoms but resurgence came after only 10 days.

Because of the likelihood of permanent untreated areas and/or recurrent collateralization, the increasing risk of extensive necrosis and the complexity and length of the varicosities owing to the constitutional agenesis of the inferior mesenteric vein, it was decided to refer the patient to the surgical team.

A surgical resection was performed 20 days after the initial diagnosis. The resection was difficult. The left colon, sigmoid and upper rectum appeared swollen and infiltrated, and had a bluish appearance evoking venous ischaemia. The mesocolon and omental appendages were bloated. A complete resection of the left descending and sigmoid colon, and upper rectum was performed and completed by loop ileostomy for protection of the low colorectal anastomosis.

## Outcome and follow-up

**Figure 6.**
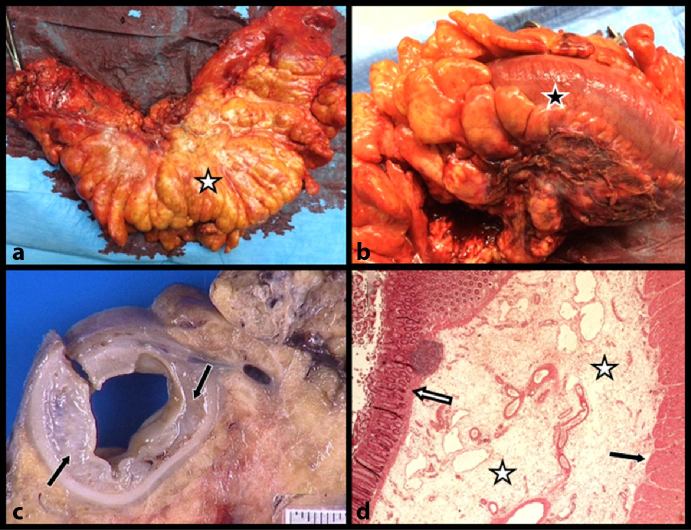
Gross anatomy of the resected specimen (a, b) reveals not only extremely diffuse congestion of the sigmoid (black star) but also of the entire mesosigmoid and the epiploic appendages (white star). Transverse section through the specimen (c) shows congestion of the mesocolon with varicosities and major submucosal oedema (black arrows). Photomicrograph (haematoxylin–eosin stain; magnification, ×25) confirmed major submucosal oedema (white stars). The mucosa itself was normal (white arrow); black arrow points to the muscular layer.

Gross anatomy of the resected specimen (Figure 6) confirmed the extremely diffuse congestion not only of the sigmoid but also of the entire mesosigmoid and the epiploic appendages. A transverse section through the specimen and histopathology showed congestion of the mesocolon with varicosities and major submucosal oedema with preservation of the mucosa itself. The post-operative period was uneventful and the protective ileostomy was closed 2 months later.

## Discussion

AVMs and arteriovenous fistulas (AVFs) of the gastrointestinal tract are often grouped together in clinical studies because their physiologic consequences are frequently exactly the same. However, there are important distinctions.^5^ Primary mesenteric AVMs are congenital and/or idiopathic and differ from secondary or acquired AVFs that are commonly caused by blunt or penetrating trauma (bullet or knife) or have iatrogenic aetiologies.^1,3–5^

In the splanchnic network, AVMs and AVFs are rather rare (about 200 cases reported). The involved arteries in decreasing order of incidence are the hepatic (45%), splenic (30%), superior mesenteric, gastroduodenal and inferior mesenteric, which is the rarest.^3,6,7^ AVMs or fistulas between the IMA and venous network are thus extremely rare.^3,8^ In an extensive review, Athanasiou et al^8^ recently identified only 26 primary or secondary cases described in the literature.

Except for rare cases related to penetrating trauma, secondary fistulas (AVFs) in the IMA territory usually occur following colonic surgery or during arterial catheterization.^3,7^ Transfixion sutures or inadvertent ligation simultaneously passing through IMA branches and veins may cause fistulas during sigmoidectomy or left hemicolectomy.^3,7,8^ Similarly, the rupture of a congenital arterial aneurysm in very close anatomic relation with a vein can also result in a secondary fistula.^3,8^ There is usually a single communication between the artery and the vein in an AVF.

Congenital AVMs result from undifferentiated embryonic vessels tail failing to regress and interconnect the arterial and venous system. They can be distinguished by a dilated feeding artery, a large tangle of vessels representing the AVM nidus with multiple arteriovenous connections and a densely opacified early draining vein or veins.^3^

In the reported case, the congenital origin of the AVM was the most probable cause. Indeed, the anamnesis of our patient was absolutely free of any previous surgical procedure or trauma. Moreover, embolization failed to successfully resolve the entity probably because multiple arteriovenous connections were present. The congenital nature of the AVM of our patient was also reinforced by the simultaneous presence of anatomic variants comprising agenesis of the inferior mesenteric vein and the presence of an exclusively mesenteric origin of the hepatic artery. Congenital malformations also often result in the formation of multiple fistulas (classical components of Osler–Weber–Rendu and Ehlers–Danlos syndromes).^3,8^

The cause of the acute clinical decompensation of the AVM in our patient is not clear. A possible worsening of cardiac fibrillation or ischaemic cardiopathy could have promoted the degradation. The history of our patient is also consistent with the classical natural history of AVMs, which, although they are congenital, do not usually clinically reveal until adulthood.^3^

The vast majority of patients with inferior mesenteric AVMs present with symptoms of portal hypertension such as variceal bleeding or ascites.^3,4,7^ Less commonly, they also present with signs of congestive IC such as abdominal pain, diarrhoea and haematochezia.^4^ However, some patients may remain asymptomatic, with only incidental detection on imaging.^3^ The intensity of symptoms is extremely variable. These symptoms are flow dependant and may range from minimal signs to severe heart failure related to left-to-right shunt.^8^

Congestive IC is one of the most serious complications (50% of cases) of inferior mesenteric AVM or AVF.^3^ The classic clinical triad includes diarrhoea, haematochezia (not present in our patient) and abdominal pain.^3^ Guidelines for the diagnostic management of IC have recently been published.^9^ A combination of modalities comprising essentially MDCT—the cardinal imaging modality for patients with suspected IC—followed by colonoscopy with biopsies for a more specific diagnosis in suspected cases constitute the recommended diagnostic sequence. Contrast-enhanced MDCT assesses the distribution and phase of IC. Secondary colonoscopy with minimal insufflation follows, except in cases of gangrene, irreversible ischaemic damage or peritonitis. A recent study recently concluded that MRI, only using pre-contrast images, could be used as a valid substitute for more invasive procedures in the diagnosis and follow-up of acute IC.^10^

Wall thickening is the most common CT manifestation of IC. The wall is usually hypodense from mural oedema. Nevertheless, wall thickening is a non-specific sign and there is a wide degree of overlap with other colonic diseases such as inflammatory bowel disease and infectious colitis. Mesenteric fat stranding and ascites are also common but non-specific signs. When ischaemia progresses to infarction, free perforation, peritonitis, pneumatosis, dilatation of the colon and portal or mesenteric gas may be seen. Direct colonoscopic visualization of a cyanotic bowel wall with submucosal oedema or haemorrhage, necrotic mucosa with ulcers, or infarction can confirm the diagnosis.

The mechanism of congestive IC in the IMA territory may be caused by the variable combination of hypoperfusion of the mucosa—owing to a steal phenomenon during which the arterial blood flow through AVM bypasses the capillary bed of the rectosigmoid—and congestive submucosal oedema due to venous hypertension—clearly diagnosed during sigmoidoscopy and confirmed by CT in our case.^1,4,5^ Acute congestive IC directly related to an AVM of the IMA territory as presented here is a very uncommon event.^1^

The congested viscera and, especially, the sigmoid colon can constitute a painful clinically palpable mass.^8^ This condition was not found in our very obese patient. Nevertheless, the sigmoid appeared extremely painful and incompressible during ultrasound examination.

Lower gastrointestinal bleeding can result from congestion of the bowel mucosa, direct fistula rupture within the lumen, necrosis owing to IC or bleeding haemorrhoids. Upper gastrointestinal bleeding resulting from oesophageal varices is rarer.

The treatment of inferior mesenteric AVMs or AVFs is complex and needs co-operation of the medical, radiological and surgical teams because the treatment needs case-specific solutions. Embolization is considered less invasive and potentially safer.^6,7^ Percutaneous endovascular arterial embolization of the feeding artery at the artery–venous junction is the technique of choice.^6 ^ It is extremely effective and has a low risk of complications in moderate-flow fistulae. Complications not only comprise serious IC but also recurrence, especially if there is more than one feeding artery. Embolization is not recommended in fistulas with large vessels because of the increased risk of extensive arterial thrombosis and ischaemia. Migration of the coils into the portal system has also been described for blood vessels of diameter >8 mm at high flow rates. Surgery would appear preferable in these situations. In our patient, two attempts at embolization failed to resolve the symptoms and extensive surgery was necessary.

## Differential diagnosis

The first retained diagnosis after emergency unenhanced abdominal MDCT was IC or inflammatory colonic disease. Colonic diverticulitis was immediately excluded because of the unusual length of the affected colonic segment and the absence of colonic diverticula. Drug-induced colitis was excluded by the anamnesis. Histopathology of the biopsies performed during optical colonoscopy confirmed a congestive and oedematous colonic mucosa with no trace of acute or chronic inflammatory process (ulcerative colitis or Crohn’s disease) and no sign of infection. Finally, contrast-enhanced abdominal MDCT and colour Doppler ultrasound were decisive for the diagnosis of the AVM causing congestive IC.

## Learning points

1. The precise aetiology of IC remains unclear in many clinical cases. Numerous occlusive or non-occlusive causes exist. In the group of occlusive aetiologies, congestive ischaemia resulting from venous congestion owing to an AVM or fistula is extremely unusual.
2. Only 26 cases of AVMs and fistulas in the territory of the IMA have been reported until 2014. IC is the most common complication with a prevalence of 50%. The classic clinical triad includes diarrhoea, haematochezia and abdominal pain.
3. Fistulas essentially develop after a surgical or an interventional radiology procedure, or trauma. On the contrary, AVMs are congenital but classically remain clinically silent until adulthood.
4. The combination of abdominal MDCT and focalized colour Doppler ultrasound was effective for the diagnosis in the reported case.
5. The treatment of these vascular entities is complex and needs case-specific solutions. Co-operation of the medical, radiological and surgical teams is then required.
